# Identification of SLAMF3 (CD229) as an Inhibitor of Hepatocellular Carcinoma Cell Proliferation and Tumour Progression

**DOI:** 10.1371/journal.pone.0082918

**Published:** 2013-12-20

**Authors:** Ingrid Marcq, Rémy Nyga, Flora Cartier, Rabbind Singh Amrathlal, Christèle Ossart, Hakim Ouled-Haddou, Hussein Ghamlouch, Antoine Galmiche, Denis Chatelain, Luciane Lamotte, Véronique Debuysscher, Vincent Fuentes, Eric Nguyen-Khac, Jean-Marc Regimbeau, Jean-Pierre Marolleau, Sylvain Latour, Hicham Bouhlal

**Affiliations:** 1 INSERM UMR925 and EA 4666 UFR de Médecine, CAP-Santé (FED 4231), Université de Picardie Jules Verne, Amiens, France; 2 INSERM U1053, Laboratoire de Physiologie du Cancer du Foie, Université Bordeaux Segalen, 146, rue Léo Saignat, Bordeaux, France; 3 Service d’hématologie Clinique et de thérapie cellulaire Centre Hospitalier Universitaire sud, Amiens, France; 4 Service de Biochimie, Centre Hospitalier Universitaire sud, Amiens, France; 5 Service d’Anatomie Pathologique, Centre Hospitalier Universitaire sud, Amiens, France; 6 Service d’Immunologie, Centre Hospitalier Universitaire sud, Amiens, France; 7 Service Hepato-Gastroenterologie, Centre Hospitalier Universitaire sud, Amiens, France; 8 Service de chirurgie digestive Centre Hospitalier Universitaire sud, Amiens, France; 9 IRNEM U768, Hôpital Necker enfants maladies, Paris, France; University Hospital Carl Gustav Carus Dresden, Germany

## Abstract

Although hepatocellular carcinoma (HCC) is one of the most common malignancies and constitutes the third leading cause of cancer-related deaths, the underlying molecular mechanisms are not fully understood. In the present study, we demonstrate for the first time that hepatocytes express signalling lymphocytic activation molecule family member 3 (SLAMF3/CD229) but not other SLAMF members. We provide evidence to show that SLAMF3 is involved in the control of hepatocyte proliferation and in hepatocellular carcinogenesis. SLAMF3 expression is significantly lower in primary human HCC samples and HCC cell lines than in human healthy primary hepatocytes. In HCC cell lines, the restoration of high levels of SLAMF3 expression inhibited cell proliferation and migration and enhanced apoptosis. Furthermore, SLAMF3 expression was associated with inhibition of HCC xenograft progression in the nude mouse model. The restoration of SLAMF3 expression levels also decreased the phosphorylation of MAPK ERK1/2, JNK and mTOR. In samples from resected HCC patients, SLAMF3 expression levels were significantly lower in tumorous tissues than in peritumoral tissues. Our results identify SLAMF3 as a specific marker of normal hepatocytes and provide evidence for its potential role in the control of proliferation of HCC cells.

## Introduction

Hepatocellular carcinoma (HCC) is one of the most incident cancers in Western populations and constitutes the third leading cause of cancer-related deaths [1]. Although the main aetiologies of HCC are now well defined, the molecular mechanisms involved in tumour initiation and progression have yet to be fully characterized. Epidemiological data suggest that the inflammation induced by chronic hepatitis B virus (HBV)/hepatitis C virus (HCV) infection and alcohol abuse are key factors in the development of HCC [2], [3]. Furthermore, imbalance between proliferation and cell death represents a tumorigenic factor in human hepatocarcinogenesis, and the observed molecular alterations in HCC are suggestive of a deregulation of apoptosis. Mutations in p53 are frequent in HCC cells and confer the latter with drug resistance [4]. Hepatocellular carcinoma cells are also insensitive to apoptosis induced by death receptor ligands such as Fas ligand FasL and tumour-necrosis-factor related apoptosis inducing ligand (TRAIL) [5], [6]. Hence, the balance between death and survival is deregulated in HCC - mainly because of over-activation of anti-apoptotic pathways [7]–[10]. Moreover, Bcl-2-family proteins play central roles in cell death regulation and are capable of regulating diverse cell death mechanisms that encompass apoptosis, necrosis and autophagy and alterations in their expression and function contribute to the pathogenesis and progression of human cancer [11]–[13]. In HCC, the observed genetic alterations lead to an imbalance in the pro- and anti-apoptotic members of the Bcl-2 family [14]. Bcl-XL is overexpressed in a great percentage of HCCs [15] and so is Mcl-1 [16]. In contrast, pro-apoptotic members of the family, such as Bax or Bcl-XS are down-regulated in HCC with dysfunction in the p53 pathway [17].

Expression and/or activation of the mitogen-activated protein kinase MAPK (RAS/RAF/ERKs) and phosphatidylinositol 3-kinases (PI3Ks)/protein kinase B (PKB-AKT)/mammalian target of rapamycin kinase (mTOR) pathways are abnormally high in many HCC cells, which render the latter resistant to apoptotic stimuli [18]–[22]. Tumour size is also positively correlated with Rapidly Accelerated Fibrosarcoma (RAF), MAPK/ERK kinase (MEK), Extracellular signal-regulated kinases (ERK) RAF/MEK/ERK activation [23]. Indeed, ERK1/2 activation is known to be an independent marker for a poor prognosis (poor overall survival (OS)) [24]. As previously reported, mTOR activation increases cell proliferation, whereas the blockade of mTOR signalling by rapamycin analogues slows tumour growth and increases survival in the HCC xenograft model [25]. These findings suggest that mTOR pathway activation has a crucial role in the pathogenesis of HCC. Furthermore, levels of the phosphorylated form of mTOR have been shown to be elevated in 15% of cases of HCC, and levels of total p70 S6 kinase (the immediate substrate for phosphorylated mTOR) are elevated in 45% of cases [26]. These data indicate that the RAF/MEK/ERK and PI3K/AKT/mTOR pathways have a major role in the pathogenesis of HCC. Hepatocellular carcinoma is a highly aggressive cancer, which is linked to chronically dysregulated liver inflammation. In fact, HCC is thought to result from persistent, non-specific activation of the immune system within the chronically inflamed liver; the resulting, repeated cycles of tissue damage, repair and regeneration are eventually followed by carcinogenesis [27], [28]. The anticancer effect of immunological synapse molecules (such as CD40-CD40L) on dendritic cells has been reported in several studies. Indeed, in the xenograft animal model, the induction of CD40 expression on dendritic cells stimulates the anti-HCC response via (i) enhancement of interleukin 12 (IL-12) production and (ii) infiltration of HCC xenografts by specific cytotoxic CD8+ T lymphocytes and natural killer (NK) cells with high production of Interferon gamma (IFNγ) [29]. However, the role of other receptors involved in immune cell stimulation and/or inhibition has not been fully tested. Here, we focused on signalling lymphocytic activation molecule family receptors (SLAMF-Rs). These receptors trigger both inhibitory and activation signals in immune cells. The SLAMF-R sub-family includes SLAMF1 (CD150), SLAMF3 (CD229), SLAMF5 (CD84), SLAMF4 (CD244/2B4), SLAMF6 (also known as NTB-A in humans and Ly-108 in the mouse), and SLAMF7 (CRACC). The SLAMF-Rs are homophilic receptors that (with the exception of SLAMF4) function as self-ligands [30]–[33]. The SLAMF-Rs’ role in modulation of the immune response depends on SLAM-associated adapter molecules (SLAM-associated protein (SAP), EWS-FLI1–activated transcript 2 (EAT-2) and EAT-2-related transducer (ERT)) [30]. Another interesting feature of SLAMF-Rs relates to the presence of one or more immunoreceptor tyrosine-based switch motifs (ITSMs) in their intracytoplasmic domains; the ITSMs recruit proteins from the signalling adaptor family, which includes SAP, EAT-2 and ERT. After binding, SAP adaptors couple the SLAMF-Rs to downstream signalling pathways. SLAMF3 is a transmembrane receptor whose expression has only been documented to date in thymocytes, T and B lymphocytes, dendritic cells, macrophages and NK cells [27]–[33]. It has been shown that T cells from Ly9-knock-out mice (Ly9 is the murine homolog of SLAMF3) proliferate poorly and produce less IL-2 after suboptimal stimulation with anti-CD3 *in vitro* [34]. In fact, ectopic expression of SLAMF3 on non-hematopoietic B16 melanoma cells triggered their killing by NK cells via SLAMF3 homophilic interactions [35]. On the basis of these observations, we sought to establish whether or not SLAMF molecules were expressed in liver tissue and to assess their involvement in hepatocyte proliferation and HCC. We first analysed the expression of SLAM molecules in hepatocytes and found that SLAMF3 was expressed by this cell type. We also observed a strong correlation between elevated SLAMF3 expression and low hepatocyte proliferation index suggesting that SLAMF3 homophilic interactions have a role in the mechanisms governing hepatocyte proliferation and the occurrence of HCC.

## Results

### Human Hepatocytes Express SLAMF3 but not Other SLAMF-Rs

**Figure 1:**
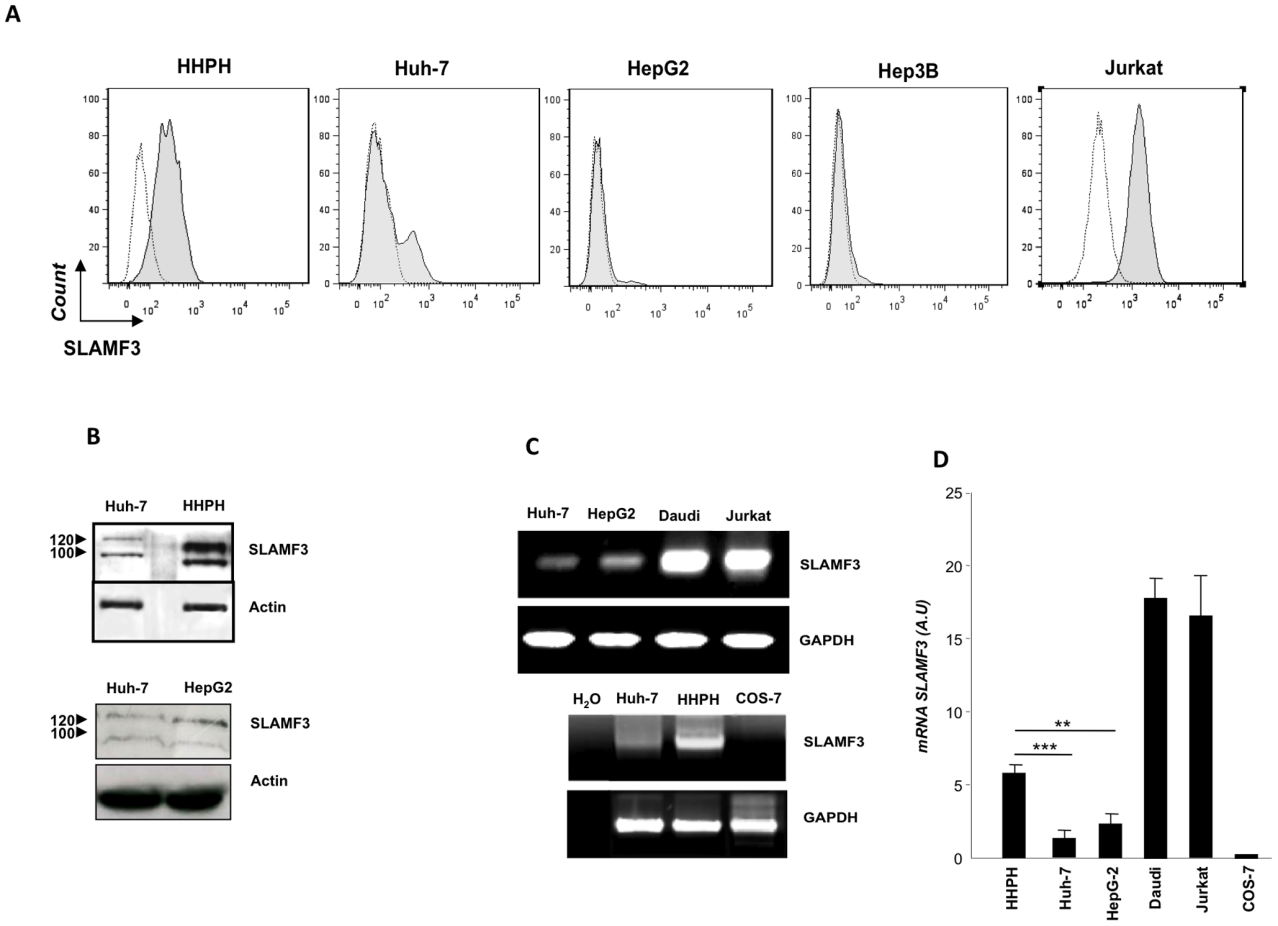
SLAMF3 is expressed by hepatocytes. (A) SLAMF3 expression was analysed by flow cytometry analysis in HHPHs and in Huh-7, HepG2 and Hep3B human HCC cell lines. SLAMF3 expression in Jurkat lymphocytes was used as a positive control; SLAMF3 staining (in grey) is overlaid by the negative control (in white) and corresponds to one representative of five independent experiments. (B) Western blot analysis of proteins extracted from Huh-7 cells, HepG2 cells or HHPHs, with a mAb (K12) against SLAMF3′s first extracellular domain (D1) or an anti-actin antibody as a control. One of four independent experiments is presented here. (C) Expression of SLAMF3 transcripts in hepatocytes. After reverse transcription, SLAMF3 cDNA was amplified by PCR using specific primers. GAPDH was amplified as a control gene and pure H_2_O was used as a PCR control. The Daudi and Jurkat human lymphocyte cell lines were used as positive controls and the monkey kidney COS-7 cell line was used as a negative control. One of three independent experiments is shown here; (D) SLAMF3 mRNA was assayed by Q-PCR in (i) HHPHs, Huh-7 cells and HepG2 cells, (ii) Daudi B lymphocytes and Jurkat T lymphocytes (positive controls) and (iii) the green monkey kidney COS-7 cell line (a negative control). Results are presented as the mean ± SD (n = 6) ***p<0.005, **p<0.01).

Firstly, a specific flow cytometry analysis indicated the expression of SLAMF3 by hepatocytes. SLAMF3 was expressed by respectively 40±5%, 15±2%, 5±2% and 4±2% of healthy human primary hepatocytes (HHPHs), Huh-7, HepG2 and Hep3B cells (with mean fluorescence intensity (MFI) ratios of 5, 2, 1.5 and 1.5, respectively). For all analyses, SLAMF3 expressing T lymphocyte cell line Jurkat was used as a positive control. Interestingly, SLAMF3 positive cells in cells from HCC cell lines (Huh-7, HepG2 and Hep3B) was weak compared to the cultured HHPHs (Figure 1A). No other members of the SLAMF-R family (i.e. SLAMF1, 2, 4, 5 and 6) were expressed by hepatocytes (Figure S1). The flow cytometry results were confirmed by Western blot analyses. The lysates of both Huh-7 cells and HHPHs showed that (i) SLAMF3 was expressed as 100 and 120 kDa proteins and (ii) SLAMF3 expression was higher in HHPHs than in the HCC (Huh-7 and HepG2) cell lines (Figure 1B). The SLAMF3 transcripts were also quantified in (i) Huh-7 and HepG2 cells, (ii) Daudi and Jurkat cells (used as positive controls) and (iii) the naturally SLAMF3-negative green monkey COS-7 cell line (used as a negative control). Relative to HHPHs, SLAMF3 transcript levels were lower in Huh-7 and HepG2 cells and higher in Daudi and Jurkat cells, supporting the results of cytometry analysis. Transcripts levels were higher in HHPHs than in Huh-7 cells (*p<0.005*) or in HepG2 cells (*p<0.01*). Expression of SLAMF3 was not observed in COS-7 cells (Figure 1 C, D).

### The Correlation between Hepatocyte SLAMF3 Expression and HCC Cell Proliferation

**Figure 2:**
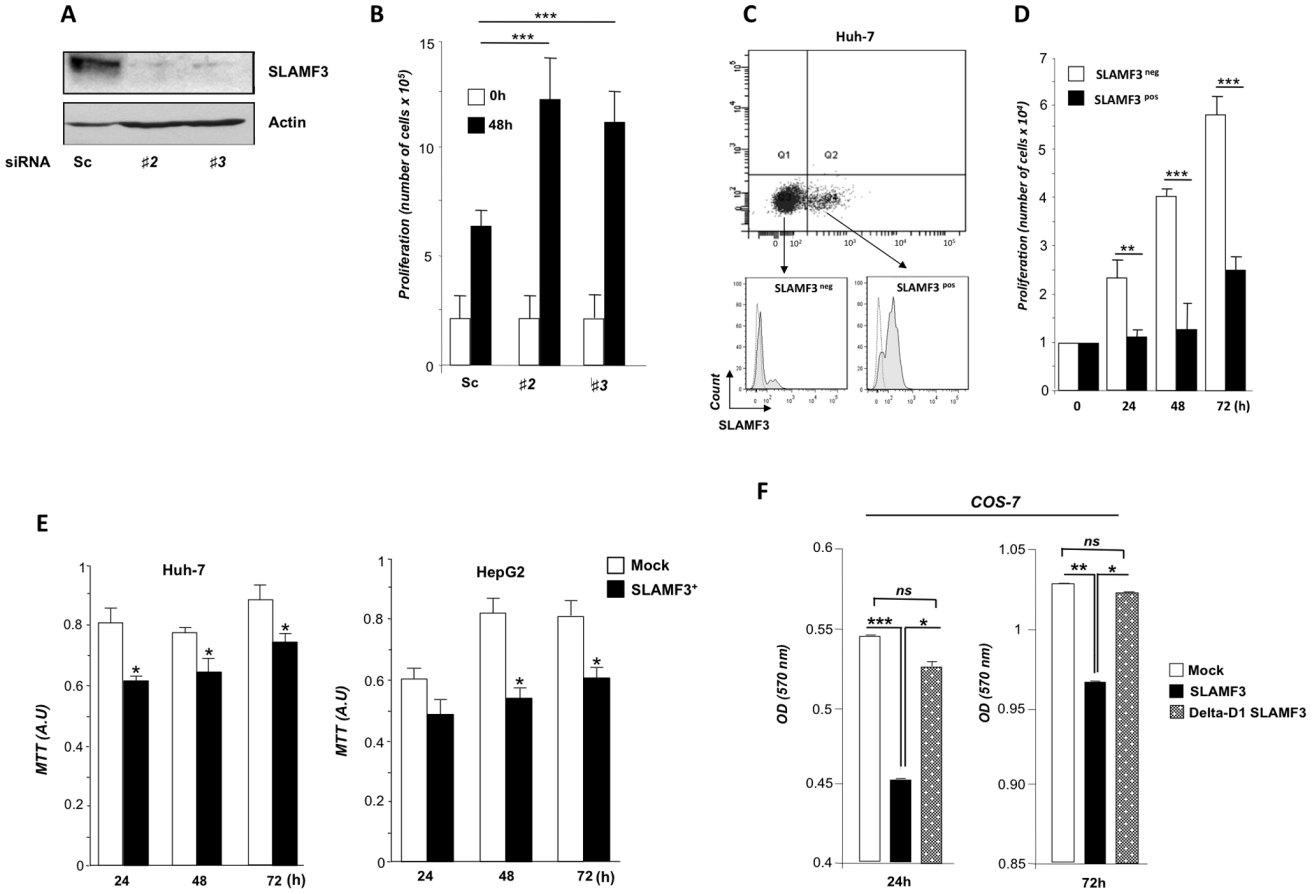
Correlation between SLAMF3 expression and HCC cell proliferation. (A) Effects of the introduction of specific siRNAs on SLAMF3 expression in Huh-7 cells. Cells were transiently transfected with a scrambled siRNA (Sc) or one of two pre-designed anti-SLAMF3 siRNAs (referred to as #2 and # 3); SLAMF3 expression was measured by Western blot analysis (anti-SLAMF3, K12). One representative of two independent experiments is shown. (B) siRNA-treated Huh-7 cells were cultured and proliferation was assayed in a Trypan blue exclusion test after 48 h. Proliferation is presented as the mean number of viable cells ± SD (n = 5; statistical significance: ***p<0.005). (C) Following our observation of bimodal SLAMF3 expression, Huh-7 cells were sorted into SLAMF3^neg^ and SLAMF3^pos^ subpopulations, as shown by the dot plot (top panel); SLAMF3 expression in the two enriched populations was measured by flow cytometry (bottom panel); (D) Huh-7 SLAMF3^neg^ and Huh-7 SLAMF3^pos^ cells were cultured separately and cell proliferation was assayed in a Trypan blue exclusion test after 24, 48 and 72 h. Proliferation is presented as mean number of viable cells ± SD (n = 4; statistical significance: **p<0.01, ***p<0.005); SLAMF3 expression was forced in Huh-7 and HepG2 cell lines and proliferation was determined in an MTT assay (E) after 24, 48 and 72 h. The results are presented as the mean ± SD (n = 4; statistical significance: *p<0.05); (F) Investigation of the role of SLAMF3′s first extracellular domain (D1) in the protein’s suppressor effect on HCC proliferation. COS-7 cells (which are naturally negative for SLAMF3) were transfected with a vector coding for wild type SLAMF3 or SLAMF3 lacking D1 (delta-D1 SLAMF3) or a mock pBud vector. Cell proliferation was monitored with MTT assays performed 24 and 72 h after transfection. The results are presented as the mean ± SD (n = 3; statistical significance: *p<0.05, **p<0.01, ***p<0.005; ns: non-significant).

Specific expression of SLAMF3 by hepatocytes and the observed difference in the SLAMF3 expression level between HCC cells and HHPHs prompted us to explore the effect of the protein on cell proliferation and apoptosis. First, we investigated the effect of SLAMF3 knockdown on HCC cell proliferation. We used the Huh-7 cell line to test the effect of SLAMF3-specific small interfering RNA (siRNA), since endogenous expression was higher in comparison to HepG2 and Hep3B cells (15±2%, 5±2% and 4±2%, respectively). SLAMF3-specific siRNAs (noted #1, #2 and #3) and a scrambled control (Sc) siRNA were introduced into Huh-7 cells and proliferation was evaluated. As shown in figure 2A, both #2 and #3 siRNAs inhibited SLAMF3 expression by up to 95% and the Sc had no effect (Figure 2A). When tested for proliferation after 48 h, the cell count had doubled in Sc siRNA treated cells whereas the cells in which SLAMF3 expression was knocked off using siRNA #2, #3 displayed higher rate (*p<0.005*) of proliferation (Figure 2B).

As shown in Fig. 1A, SLAMF3 expression was observed only in 15±2% of Huh-7 cells. Given this bi-modal expression pattern, SLAMF3-negative (Huh-7 SLAMF3^neg^) and SLAMF3-positive (Huh-7 SLAMF3^pos^) sub-populations were sorted and expression of SLAMF3 was analysed by flow cytometry. After sorting, 90% of Huh-7 SLAMF3^pos^ cells and 10% of Huh-7 SLAMF3^neg^ cells expressed SLAMF3 (Figure 2C). The SLAMF3 expression was stable in each sub-population until 48 h after sorting (data not shown). When the sorted sub-populations were cultured separately and evaluated for proliferation, SLAMF3^pos^ cells were found to have a significantly lower proliferation rate than SLAMF3^neg^ cells cultures did (*p<0.01* at 24 h; *p<0.005* at 48 and 72 h) (Figure 2D).

To confirm the inhibitory effect of high levels of SLAMF3 expression on cell proliferation, we transiently transfected Huh-7 and HepG2 cell lines with either an empty (mock) vector or an expression vector coding for SLAMF3. After transfection, SLAMF3 expression was respectively 20-fold and 13-fold higher in Huh-7 and HepG2 cells than in control experiments (Figure S2). The results of a (3-(4,5-dimethylthiazol-2-yl)-2,5-diphenyltetrazolium bromide (MTT) assay showed that SLAMF3 over-expression significantly (*p<0.05)* inhibited Huh-7 and HepG2 proliferation when evaluated at 24, 48 and 72 h (Figure 2E). This result was confirmed by carboxyfluorescein succinimidyl ester CFSE staining. Interestingly, when SLAMF3^pos^ and SLAMF3^neg^ cell fractions were gated and analysed in terms of the proliferation index, we observed that CFSE staining was lower in SLAMF3^neg^ cells than in SLAMF3^pos^ cells - confirming the strong correlation between high SLAMF3 expression and low cell proliferation (Figure S3). The homophilic interactions between SLAMF3 molecules occurs through the extracellular V-like domain 1 [36]. In order to assess this domain’s involvement in SLAMF3′s anti-proliferative role, we designed a SLAMF3 mutant lacking the first V-like domain (delta-D1-SLAMF3). To avoid interference from endogenous expression, these experiments were performed on COS-7 cells, which do not produce native SLAMF3 (see Fig. 1 C). The cells were transfected with either delta-D1-SLAMF3, wild type (SLAMF3) or mock vector and their proliferation was evaluated. Introduction of delta-D1-SLAMF3 resulted in much weaker inhibition of proliferation than introduction of wild type SLAMF3 did (Figure 2F).

### High Levels of SLAMF3 Expression Inhibit Cell Motility

**Figure 3:**
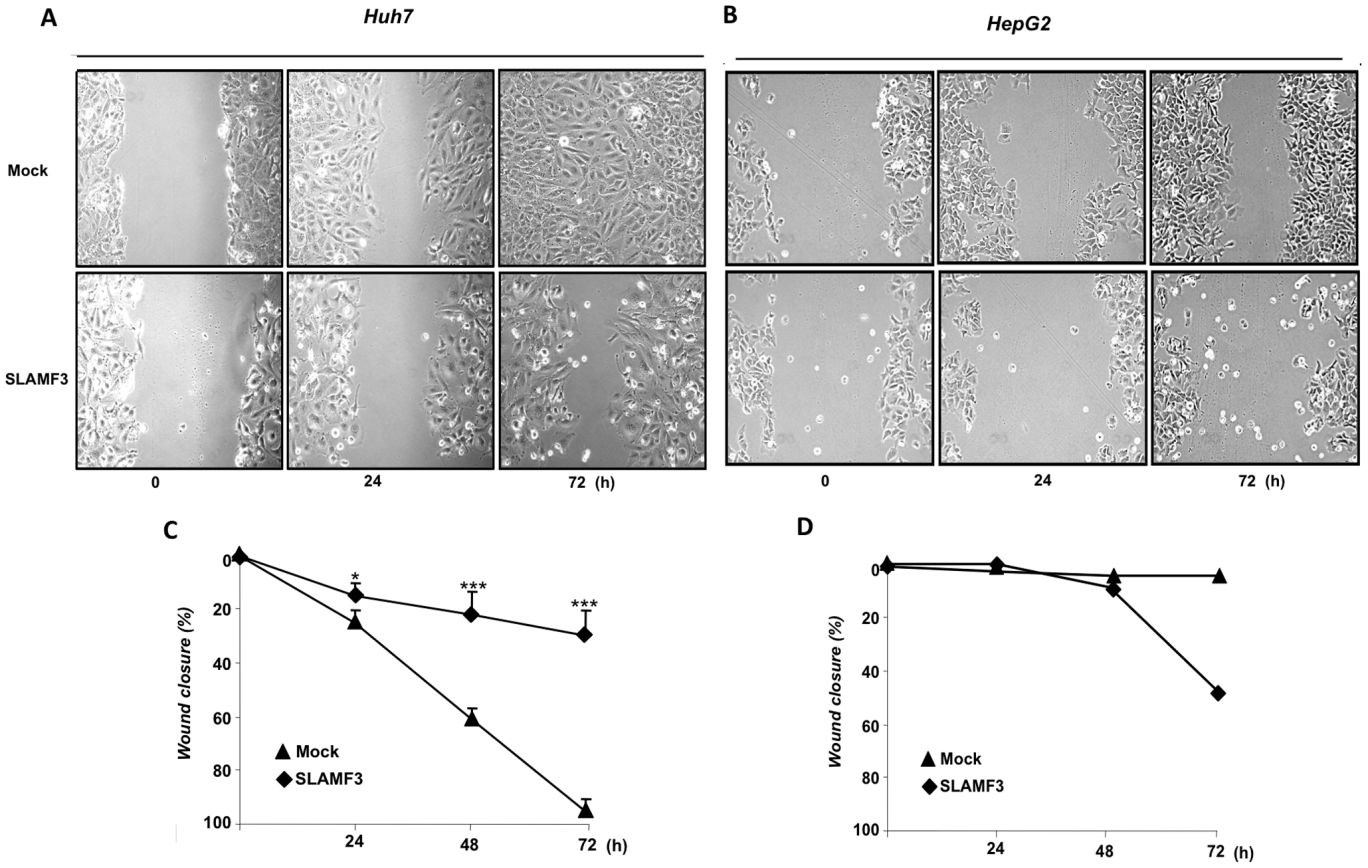
Correlation between HCC cell SLAMF3 expression and cell motility. Cell migration activities in Huh-7 (A) and HepG2 (B) cells overexpressing SLAMF3 and in mock cells were compared in a wound-healing assay. Same areas of culture plate were photographed at the indicated time points. The migratory index corresponds to the percentage of wound closure (estimated using Image J software) and presented as the mean ± SD from three independent experiments with Huh-7 cells (C) (statistical significance: *p<0.05 ***p<0.005) and from one experiment for Hep3B cells (D).

By using wound-healing assays, we next showed that over-expression of SLAMF3 in HCC cells resulted in substantial changes in cell shape (a smooth leading edge, with few lamellipodia). In contrast, control cells appeared to be flatter and more irregular, with many lamellipodia at the leading edge (suggestive of a migratory cell phenotype) (Figure 3A, B). The results of wound healing assays revealed that SLAMF3-over-expressing cells were much less motile than control cells, which resulted in the non-colonization of areas that were completely confluent in mock experiments (Figure 3C, D); p<0.05 at 24 h and p<0.005 at 48 and 72 h). In Huh-7 cultures, we used confocal microscopy to assess the organization of actin filaments after phalloidin staining. We observed that SLAMF3^neg^ cells had stress fibres at the leading edge, whereas the bundles of stress fibres in SLAMF3^pos^ cells did not have a preferred orientation suggesting a less motile phenotype (Figure S4).

### High Levels of SLAMF3 Expression Inhibit the MAPK ERK1/2, JNK and mTOR Pathways and Enhance Apoptosis

**Figure 4:**
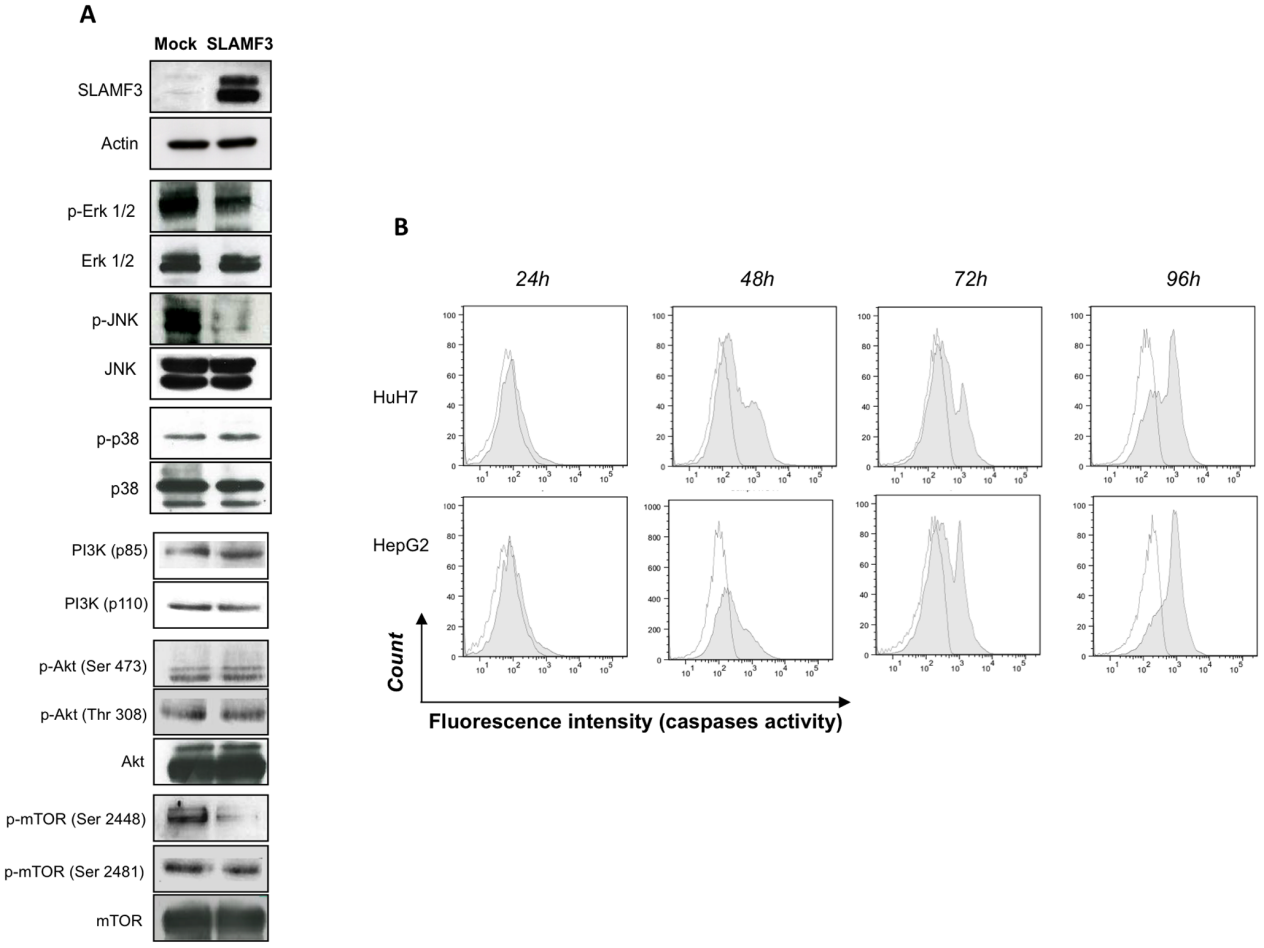
Restoration of SLAMF3 expression in HCC cells inhibits MAPK ERK1/2, JNK and mTOR pathways and induces caspase-dependent apoptosis. (A) SLAMF3 expression was confirmed by Western blot analysis in SLAMF3-over-expressing Huh-7 and mock cells at 48 h post-transfection. ERK1/2, JNK, p38, AKT and mTOR proteins were detected as controls. The activation levels of MAPK ERK1/2 (Thr 202/Tyr 204), JNK (Thr 183/Tyr 185), p38 (Thr 180/Tyr 183), PI3K (p85/p110 Thr 467/Tyr 199), AKT (Ser 473, Thr 308) and mTOR (Ser 2448, Ser 2481) are represented. One of three representative experiments is shown here. (B) The results of an active caspase assay based on cell-permeable fluorochrome inhibitor of caspases (FLICA). Caspase activity was evaluated in HCC cells (Huh-7 and HepG2 lines) at the indicated times after the ectopic introduction of SLAMF3 vector. Caspase activity is shown in the SLAMF3^neg^ subpopulation (in white) and the SLAMF3^pos^ (overlaid in grey) subpopulation from one representative of two independent experiments.

As mentioned above, RAF/MEK/ERK and PI3K/AKT/mTOR pathways have a major role in the pathogenesis of HCC. To assess the effect of high SLAMF3 expression on HCC proliferation and signalling pathways, we evaluated the phosphorylation status of the major protein of MAPK and PI3K/AKT/mTOR pathways in Huh-7 cells over-expressing SLAMF3. We found that the restoration of high SLAMF3 expression specifically inhibited the phosphorylation of ERK1/2 (Thr 202/Tyr 204) and N-terminal kinases JNK (Thr 183/Tyr 185) but did not affect p38 activation. Furthermore, high SLAMF3 expression decreased mTOR phosphorylation specifically on serine 2448 but did not influence phosphorylation of serine 2481. No changes in PI3K and AKT phosphorylation were observed (Figure 4A).

To establish whether inhibition of proliferation induced apoptosis, cells over-expressing SLAMF3 were assayed for apoptosis via annexin V (AV) and 7-aminoactinomycin D (7-AAD) staining 48 h after the introduction of SLAMF3. Where 7% of mock- transfected cells stained positive for AV and 7-AAD (i.e. spontaneous apoptosis), this percentage was significantly higher (20%, *p<0.005*) in cells overexpressing SLAMF3 (Figure S5A, B). In SLAMF3-transfected cells, the percentage of AV/7-AAD-positive cells was significantly higher (*p<0.01*) in SLAMF3^pos^ populations than in SLAMF3^neg^ populations (Figure S5C). In order to check whether the observed apoptosis was caspase-dependent, we performed a fluorochrome inhibitor of caspases (FLICA) assay for active caspases. The results showed that overexpression of SLAMF3 in Huh-7 and HepG2 cells induced the activation of caspases (Figure 4B). The involvement of the Bcl-2 family pathway was also studied; only a slight enhancement of BAD expression was observed, whereas no significant change was seen for BclXL (Figure S5 D) and other tested members (data not shown).

### SLAMF3 Expression Inhibits Tumour Growth in Nude Mice Xenografted with Human HCC Cells

**Figure 5:**
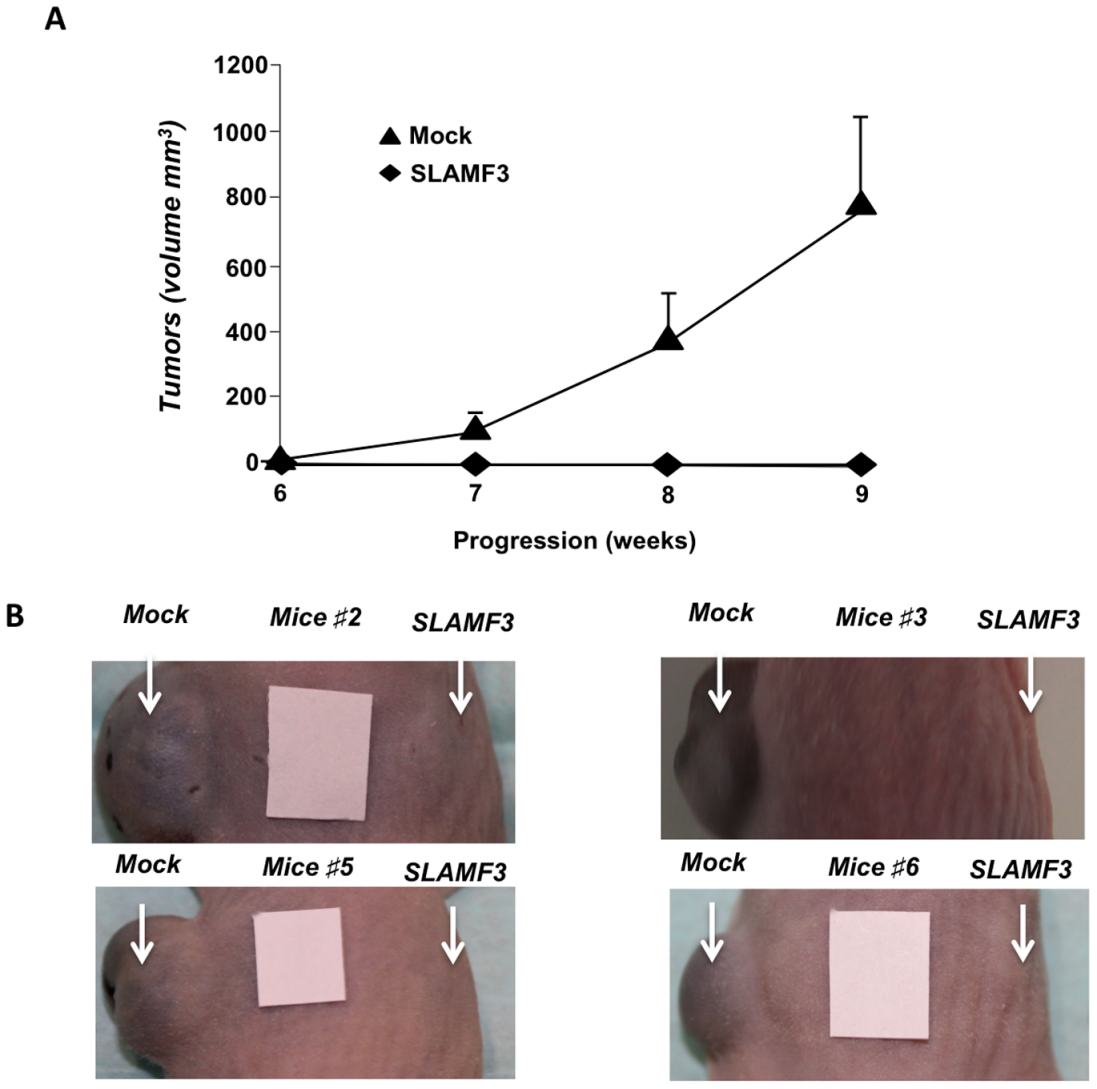
The effect of SLAMF3 expression on the growth of HCC xenografts in nude mice. (A) Tumour volumes were measured 6, 7, 8 and 9 weeks after injection in nude mice. Four out of ten animals developed a tumour on the left flank (after injection of mock-transfected Huh7 cells) but none of the ten developed a tumour on the right flank (after injection of SLAMF3-overexpressing Huh7 cells) and represented as mean ± SD (*p<0.05); (B) photographs of tumour progression on left flanks (mock-transfected Huh7 cells) and right flanks (SLAMF3-overexpressing Huh7 cells) (mice #2, #3, #5 and #6).

To further confirm the inhibitory effect of SLAMF3 on HCC cell proliferation and the induction of apoptosis, we studied xenograft growth after the injection of mock Huh-7 cells (left flank) and SLAMF3 overexpressing Huh-7 cells (right flank) into Nude mice. Of the ten animals injected with Huh-7 cells, four (40%) developed left-flank tumours within six weeks of injection. However, none of the flanks injected with SLAMF3-overexpressing Huh-7 cells developed tumours prior to sacrifice at 10 weeks (Figure 5A, B).

### SLAMF3 Expression is Repressed in Resected Tumour Samples from HCC Patients

**Figure 6:**
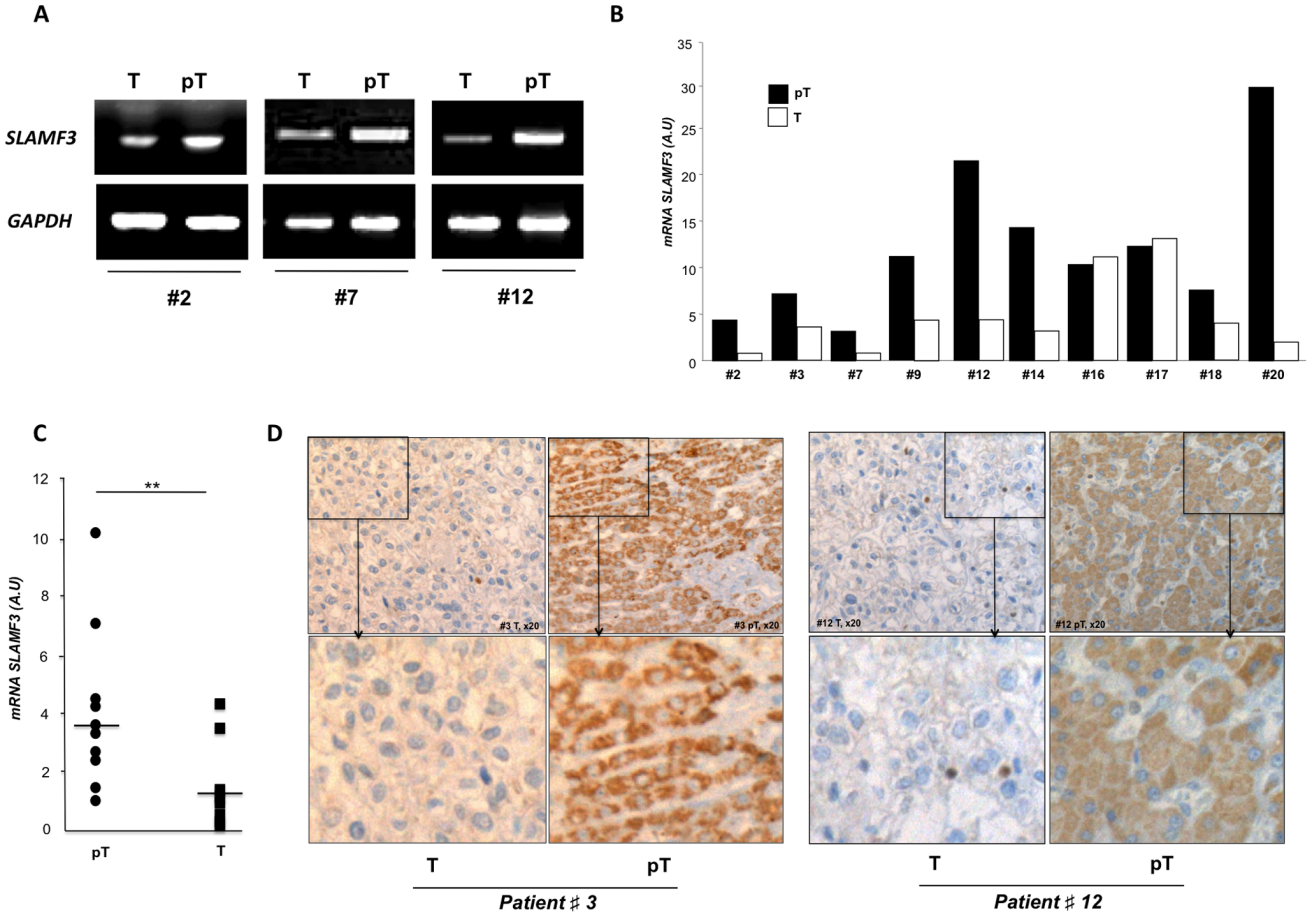
SLAMF3 expression is repressed in tumour cells from ten resected HCC patients. (A) SLAMF3 mRNA expression was analysed in hepatocytes from resected HCC patients. Specific amplification of SLAMF3 mRNAs using PCR (representative patients #2, #7 and #12) and quantitative PCR in tumour tissues (T) and peritumoral tissues (pT) presented separately (B) and as median (**p<0.05) (C); (D) Immunohistochemical staining of SLAMF3 in T and pT areas from representative patients #3 and #12 (×20).

The marked difference in SLAMF3 expression between normal and cancerous hepatocytes observed in cell culture prompted us to check for this difference in tumour samples from HCC patients. We analysed SLAMF3 expression in primary tumours (T) and peritumoral (pT) tissues obtained from 10 HCC patients (mean ± SD age: 65.7±9.7 yrs) undergoing liver tumour resection (mean ± SD tumour size: 4.8±2 cm) in our Department of Surgery. Other clinical characteristics of the resected patients are summarized in Table S1. One of the 10 patients was seropositive for HCV and another was seropositive for HBV. Total mRNA was extracted and SLAMF3 transcripts were quantified. SLAMF3 mRNA was found to be expressed in all T and pT samples (Figure 6A, B). Interestingly, SLAMF3 mRNAs levels were significantly lower in T samples than in pT tissues in 8 of the 10 patients, whereas no significant T vs pT difference was observed in the 2 remaining patients (#16 and #17) (Figure 6C; *p<0.01*). These findings confirmed our results obtained in cell lines and cultured HHPHs. We also observed the low SLAMF3 at the protein level in immunohistochemical staining experiments, as shown for representative samples from patients #3 and #12 in Figure 6D.

## Discussion

In the present work, we showed for the first time that hepatocytes express SLAMF3 and provided evidence of the protein’s involvement in the progression of HCC (i.e. loss of its expression in cancerous cells). We also showed that mRNA and protein levels of SLAMF3 are significantly lower in HCC cell lines than in HHPHs. This difference was confirmed in tumour samples from HCC patients. The link between SLAMF3 expression and proliferation was demonstrated *in vitro* and then validated by the inhibition of HCC progression in Nude mice xenografted with SLAMF3-overexpressing HCC cells. It was recently reported that SLAMF3 has a similar role in lymphocytes; in contrast to SLAMF1 and SLAMF6, SLAMF3 has a negative effect on the signalling pathways required for innate-like lymphocyte development in the thymus [37]. The observed effect may be attributed to both decrease in the proliferation of cells over-expressing SLAMF3 and the induction of apoptosis. In the present work, we also observed an association between restoration of SLAMF3 expression in HCC cells and the significant inhibition of ERK and JNK phosphorylation, which are constitutively activated in HCC and associated with the malignant HCC phenotype [23], [24]. Other studies using *in vivo* HCC animal models and human HCC tissue specimens have evidenced greater MAPK ERK expression and activity in tumours relative to the surrounding tissue [38], [39]. Indeed, ERK activity has clinical relevance since it positively correlated with tumour size and aggressive tumour behaviour and is considered to be an independent prognostic marker for poor overall survival (OS) [24]. In human T cells, SLAMF3 engagement attenuates T-cell receptor signalling and reduces ERK activation. Murine T cells lacking SLAMF3 exhibit low Th2 responses [34], [40]. The JNK pathway is known to be a negative regulator of the p53 tumour suppressor and its role in cell survival is well established [41]. Based on the correlation between elevated JNK kinase activity and tumour cell proliferation, it has been suggested that JNK has an oncogenic role [42]. In contrast, reports of low p38 activity in HCC (relative to non-tumorous tissues) suggest that elevated p38 MAPK activity induces apoptosis in hepatoma cell lines [43]. The members of the BCL2 family can function both as positive or negative regulators of apoptosis. Changes in BCL2 family expression and/or activation have been observed in several tumour types [44]–[47]. Indeed, expression levels of BCLXL (an anti-apoptotic factor) are elevated in HCC [48]. Furthermore, a recent report indicated that BID (a pro-apoptotic member) is down-regulated in a subset of HCCs in the context of viral hepatitis [49]. The pro-apoptotic BAD reportedly exert an important regulatory role in cell death in normal liver cells. Concordantly, BAD expression is low in HCC. It was recently reported that sorafenib increases the expression of BAD and thereby sensitizes HCC cells to apoptosis [50]. In our present study, the restoration of high SLAMF3 expression in HCC cells produced a minor enhancement of BAD levels but did not have an effect on BCL-XL. Taken together with the fact that p38 phosphorylation was not modified, our results suggest that SLAMF3 expression in hepatocytes (i) primarily controls cell proliferation through MAPK, ERK and JNK inhibition and (ii) induces apoptosis through caspase pathway. However, additional experiments are required to pinpoint the involvement of BCL2 family members in SLAMF3-dependent apoptosis.

Furthermore, it has been firmly established that constitutive activation of the PI3K/AKT/mTOR signalling pathway is a determinant of tumour cell growth and survival in many different solid tumours [51]. This pathway can be hyper activated by enhanced stimulation of receptor tyrosine kinases such as the insulin-like growth factor receptor (IGF-R) and epidermal growth factor receptor (EGF-R). EGF receptor in particular whose expression is upregulated in HCC and human cirrhotic liver points out towards hyperactivation of PI3K/AKT/mTOR pathway in both conditions [52]. As reported elsewhere, mTOR activation increases cell proliferation, whereas the blockade of mTOR signalling by rapamycin analogues slows tumour growth and increases survival in the HCC xenograft model. These findings suggest that mTOR pathway activation has a crucial role in the pathogenesis of HCC [53], [54]. Based on this observation we investigated the link between effect of SLAMF3 expression on proliferation and activation of mTOR and found that SLAMF3 expression had an inhibitory effect on mTOR phosphorylation (Ser 2448) in a PI3K- and AKT-activation-independent manner. The restoration of higher SLAMF3 expression in HCC cells may indirectly control the activation of mTOR by inhibiting Erk phosphorylation without affecting the PI3K/AKT pathways. Furthermore, it has also been reported that mTOR is activated through the ERK pathway as well as through the AKT pathway. The effector kinases in the mTOR pathway activation are AKT, ERK1/2 and ribosomal S6 kinase RSK1, which phosphorylates and inactivates tuberous sclerosis complex 1/2 (a tumour suppressor) and activates mTOR on serine 2448 [55]. The molecular partners linking SLAMF3, ERK/JNK and mTOR have yet to be identified in hepatocytes. Interestingly, restoration of SLAMF3 expression in HCC cells reduced cell migration and the rearrangement of cytoskeletal elements, a phenomenon associated with the promotion of metastasis and the migration of neoplastic cells [56].

SLAMF3 comprises of four extracellular Ig-like domains. Domains 1 and 3 are very similar, as are domains 2 and 4 [57], [58]. Domain 1 is involved in homophilic interactions. We therefore tested the anti-proliferative effect of a SLAMF3 mutant lacking domain 1 in a COS-7 cell line (in order to minimise the residual effect of endogenous SLAMF3). We showed that the absence of domain 1 significantly abrogated the anti-proliferative effect of SLAMF3. Taken as a whole, our results suggest that homophilic interactions between SLAMF3 molecules on adjacent hepatocytes trigger a proliferation-inhibiting signal. It is noteworthy that SLAMF3 binds to the µ-2 chain of the AP-2 adaptor complex and is the only member of the SLAM family capable of being internalized by clathrin-mediated endocytosis [40], [59]. Our data suggest that in healthy hepatocytes, SLAMF3 may be either expressed or internalized, and therefore allows or inhibits cell proliferation. The factors that control hepatocyte SLAMF3 expression and/or internalization remain to be identified. Our results show that SLAMF3 expression in HCC promotes the proliferation of tumour cells. However the molecular mechanisms underlying the downregulation of SLAMF3 have yet to be identified.

## Methods

### Patients and Primary Biological Samples

Ten pairs of tumour (T) samples and matched peritumoral (pT) samples were obtained from HCC patients undergoing surgical resection at Amiens University Hospital (Amiens, France). Our protocol was approved by the local independent ethics committee (Comité de Protection des Personnes (CPP) Nord-Ouest, Amiens, France). Patients were provided with information on the study procedures and objectives and all gave their written consent to participation. Total RNAs were extracted from tissue samples (using an RNeasy Mini Kit (Qiagen, Courtaboeuf, France)) and reverse-transcribed (using a High Capacity cDNA Reverse Transcription kit and random hexamer (Applied Biosystems, Saint Aubin, France)) prior to the specific quantification of SLAMF3 mRNA.

### Cell Culture and Reagents

Two human HCC-derived cell lines (Huh-7 and HepG2) and the monkey kidney cell line COS-7 were purchased from ATCC (Molsheim, France). Cells were maintained in DMEM (Life Technologies/Invitrogen, Saint Aubin, France) supplemented with 10% foetal calf serum (FCS) (PAA, Velizy-Villacoublay, France), 1% penicillin/streptomycin. Cultured HHPHs were obtained from 7 different donors and were maintained in serum- and phenol-red-free HBC™ Basal Medium (Lonza, Basel, Switzerland). Daudi B lymphocytes and Jurkat T lymphocytes (both from ATCC) were maintained in RPMI 1640 (Institut de Biotechnologies, France) or MDM supplemented with 10% FCS, 1% penicillin/streptomycin and L-glutamine.

### Antibodies and Reagents

Unconjugated monoclonal anti-SLAMF3 HLy9.1.25 antibody was a gift from Dr P. Engel. Fluorescein isothiocyanate (FITC)- and phycoerythrin (PE)-conjugated monoclonal anti-SLAMF3 (clone HLy9.1.25) was purchased from AbD Serotec (Colmar, France). Goat polyclonal anti-SLAMF3 (K-12) was obtained from Santa Cruz Biotechnology (Heidelberg, Germany). Phycoerythrin-conjugated anti-SLAMF2 (CD84), -SLAMF4 (CD244, clone 2-69) and -SLAMF1 (CD150, clone A12) and FITC-conjugated anti-mouse IgG1 κ isotype control (clone MOPC-21) came from BD Pharmingen (Le Pont de Claix, France). Phycoerythrin-conjugated anti-SLAMF6 (NTB-A, clone NT-7) was from Biolegend (OZYME, Saint Quentin en Yvelines, France) and PE-conjugated anti-mouse IgG1 was bought from Beckman Coulter (Villepinte, France). Rabbit antibodies directed against human ERK, phospho p44/42 MAPK ERK1/2 (T202/Y204), PI3K p85 (T467), PI3K p110 (Y199), phospho-AKT (Ser 473), phospho-AKT (Thr 308), AKT, phospho-mTOR (Ser 2448), phospho-mTOR (Ser 2481) and mTOR and HRP-conjugated anti-rabbit antibodies were purchased from Cell Signalling Technology (Beverly, MA, USA). Mouse anti-phospho-JNK (T183/Y185) and anti-JNK antibodies and HRP-conjugated anti-goat antibodies came from Santa Cruz Biotechnology (Santa Cruz, CA, USA) and HRP-conjugated anti-mouse antibody was purchased from Amersham Pharmacia Biotech (Orsay, France). Anti-BAD and anti-BclXL antibodies were purchased from Cell Signalling Technology. Rhodamine-conjugated phalloidin (a gift from Dr R. Mentaverri in the Pharmacology Laboratory, Jules Verne University of Picardie, Amiens, France) was used at a dilution of 1/50 to stain actin filaments. The MTT assay kit was from Promokine and the 5(6)-carboxyfluorescein diacetate *N*-succinimidyl ester (CFSE) dye came from Sigma-Aldrich (St Quentin Fallavier, France).

### mRNA Extraction, Quantitative PCR, Sequencing and Plasmid Construction

Total mRNA was extracted with an RNeasy kit (Qiagen) and RT-PCR was performed using 100 ng of total RNA. Quantitative PCR was performed according to the Taqman Gene Expression protocol (Applied Biosystems) using the following primers for SLAMF3: forward 5′- TGGGACTAAGAGCCTCTGGAA a-3′, reverse 5′-ACAGAGATTGAGAACGTCATCTGG-3′ and MGB probe with 6-FAM (5′-CCCCAACAGTGGTGTC-3′). The transcription of GAPDH, as control, was measured as an endogenous housekeeping control. For hepatic SLAMF3 sequencing, PCR products (from Huh-7, Hep-G2, Daudi and Jurkat lymphocytes) were purified and cloned into a TOPO TA Vector (Invitrogen, #45-0641) for sequencing. Briefly, after a purification step, the sequencing reaction (25 cycles) was performed using BigDye Terminator 3.1 (Applied Biosystems), F-M13 and R-M13 and PCR primers, as previously described (with the TOPO TA Vector kit). Purified products were sequenced and analysed on an AB Prism 7100 system running the appropriate software (Applied Biosystem). Sequence alignment and comparison were performed using BLAST and Ensembl Genome Browser (http:/blast.ncbi.nlm.nih.gov; www.ensembl.org). Hepatic SLAMF3 was cloned into a pBud CE4.1 plasmid. For SLAMF3 lacking the first extracellular domain (delta-D1 SLAMF3), the following primers were used: forward 5′- ATGGTCACCATGAAGTCTGTG-3′, reverse 5′-CTCATGGGACTAAGAGCCTCT-3′. delta-D1 SLAMF3 was cloned into a pBud CE4.1 plasmid. For SLAMF3 over-expression, cells (0.3×10^6^) were first seeded into six-well plates 24 h prior to transfection. The cells were transfected with 0.8 µg of plasmid DNA using the FuGENE HD Transfection Reagent Kit (Roche, Meylan, France) according to the manufacturer’s instructions. Cells were incubated for 48 h at 37°C before analysis of SLAMF3 expression by mRNA quantification, flow cytometry and Western blot.

### Hepatocyte SLAMF3 Knock-down by siRNA and Transfection

Three specific siRNAs (Ly9.1, Ly9.2 and Ly9.3, referred to as #1, #2 and #3; ID114219 (Ly9-1): 5′-CCAAGUGGAGUUACUCCCUtt-3′, ID212885 (Ly9-2): 5′-CGUCCCAAAGAAAAUGUAAtt-3′, ID106775 (Ly9-3): 5′-GGAAUUCACCCUGUUCGUCtt-3′, respectively) and the scrambled control (Sc) siRNA were predesigned by Ambion® and introduced into Huh-7 cells using the Silencer® siRNA Transfection II Kit (Applied Biosystems, Ambion®, Saint Aubin, France) according to the manufacturer’s instructions. Briefly, 2×10^5^ cells were seeded in 6-well plates and then incubated at 37°C for 50 h with siRNA (60 nM) in the presence of NeoFX solution (5 µL) in a final reaction volume of 100 µL of OptiMEM medium (Life Technologies/Invitrogen). The kit included positive and negative siRNA controls. The positive siRNA control targeted the GAPDH gene and the negative control siRNA was a scrambled sequence that shared no homology with the human, mouse or rat genomes. Expression was checked by Western blot analysis.

### Western Blot Analysis

Cells (10^6^ per assay) were lysed in Nonidet P40 (NP40) buffer (1% NP40, 50 mM Tris pH 7.5, 10% glycerol, 150 mM NaCL, 1 mM EDTA, 100 mM Na_3_VO_6_, 0.5 mM phenylmethanesulphonyl fluoride (PMSF), 5 mg/ml aprotinin, 5 mg/ml leupeptin and 2 mg/ml pepstatin) containing protease and phosphatase inhibitors (Roche). Equal amounts of each protein sample were separated by electrophoresis on SDS-PAGE, blotted onto nitrocellulose membranes (Bio-Rad, Munich, Germany) and blotted with antibodies against SLAMF3 (K12), actin (C-11) and other tested molecules. Blots were developed with the enhanced chemiluminescence (ECL) system (Amersham Pharmacia Biotech).

### Flow Cytometry Analysis

Cells were collected in cold PBS/0.01% sodium azide/0.5% BSA, washed and then incubated with fluorescent-conjugated primary or isotype-matched antibodies for 20 min at 4°C. Following extensive washing (in PBS/0.01% sodium azide), cells were fixed (in 1% paraformaldehyde) and 5,000 viable events were analysed (on a FACSAria machine running FACSDiva software; BD Biosciences, Le Pont de Claix, France). Results were expressed as the percentage of positive cells and the MFI ratio (the MFI obtained in the presence of specific antibody divided by the MFI obtained with a non-specific, matched isotype).

### Immunohistochemistry

Slides of whole sections were dewaxed in xylene and rehydrated in graded alcohol dilutions. Endogenous peroxidase activity was blocked by incubation in H_2_O_2_ for 2×15 minutes. Antigen retrieval consisted of microwave processing at 750 W and 150 W for 15 minutes each and then pressure cooking in 0.01 M citrate buffer (pH 6.0). The slides were incubated with an anti-SLAMF3 monoclonal antibody (clone HLy1.9.25; 1/50 dilution) and the matched isotype antibody as a control. Antibody binding was detected after 30-minute incubation with a biotinylated anti-mouse secondary antibody and then 20-minute incubation with HRP-labelled streptavidin. The bound antibody was detected with a Super Sensitive Detection kit (Biogenex, Microm Microtech, Francheville, France). The slides were counterstained with haematoxylin.

### Hepatocyte Proliferation, Migration and Motility Assays

For the MTT assay, cells (Huh-7, HepG2, mock and SLAMF3-overexpressing cells) were seeded at 10^4^ cells/well in 96-well plates. At 24, 48 and 72 h, cells were rinsed and exposed for 1 h to a solution of thiozalyl blue tetrazolium bromide suspended at a concentration of 0.5 mg/ml in colourless culture medium. Reduced purple formazan crystals were extracted with DMSO and analysed at a wavelength of 560 nm. Cell proliferation at 24, 48 and 72 hours was also determined by flow cytofluorography with 5 µmol/L CFSE staining (Sigma-Aldrich) and a Trypan blue exclusion test. In some experiments, COS-7 cells were transfected with wild type SLAMF3 or delta-D1 SLAMF3 vectors and processed essentially as described above. Cell migration was assessed using a wound healing assay. Briefly, 90%-confluent monolayers of Huh-7 or HepG2 cells were scratched with a sterile micropipette tip and then washed to remove floating cells and debris. The cells were cultured in complete medium and wound closure was photographically monitored at 0, 24 and 72 hours. For phalloidin staining, cells were incubated with FITC-conjugated anti-SLAMF3, fixed and permeabilized (Cytofix/Cytoperm Fixation/Permeablization Kit; BD Pharmingen). Cells were incubated with rhodamine-conjugated phalloidin and the immunostained sample was examined under a confocal microscope (LSM710, Zeiss, Le Pecq, France).

### Cell Death Assays

For the apoptosis assay, the PE-AV/7-AAD kit (BD Pharmingen) was used according to the manufacturer’s instructions. Mock and SLAMF3-over-expressing hepatocytes (Huh-7 or HepG2) were incubated at 37°C for 24, 48, 72 and 96 h after transfection with the cell membrane-permeant, fluorescent inhibitor-based FLICA probes (FAM-VAD-FMK, FLICA CaspaTag, Chemicon, Merck Millipore, Darmstadt, Germany) diluted in PBS. The probes form covalent bonds with activated caspase enzymes and are therefore trapped within cells containing active caspases (a hallmark of apoptotic cells). Probe uptake was measured by flow cytometry.

### Growth of Human HCC Xenografts in Nude Mice

Animal experiments were carried out in accordance with European Guidelines for the care and Use of Animals for Research Purposes. After obtaining approval from our local Institutional Animal Care and Use Committee (Comité Régionale d’Ethique en Matière d’Expérimentation Animale CREMEA-protocol number 241012-15; Université Jules Verne Picardie, Amiens, France), ten 5-week-old male Balb/c nude mice (Janvier, Le Genest Saint Isle, France) were used for HCC xenograft experiments, as previously described [60]. Twenty-four hours after transfection, SLAMF3-over-expressing Huh-7 cells or matched control (mock) Huh-7 cells (3×10^6^ in 0.20 ml) were subcutaneously injected into the animals’ left (mock) and right (SLAMF) flanks. Mice were housed in our animal facility under specific pathogen-free conditions. Once a tumour had appeared (4–6 weeks after cell injections), its diameter was measured weekly using callipers. Animals were euthanized by cervical dislocation at week 10 post-injection or when the tumour volume >1.5 cm^3^). To ameliorate suffering, mice were kept 5 per cage max. The animals were sacrificed as soon as the tumour reaches a volume of 1 cm^3^. Tumour volumes were calculated according to a standard equation (width^2^×length/2). In each group, the mean ± SD tumour volume (mm^3^) was plotted against time (weeks).

### Statistical Analysis

Unless otherwise stated, results are expressed as the mean ± SD. Statistical analyses (Mann-Whitney tests and an analysis of variance) were performed with Prism software (version 4.0, GraphPad Inc., San Diego, CA, USA). In the xenograft experiments, Fisher’s exact test was used to assess differences in mean ± SD tumour area between control (mock) cells and SLAMF3-over-expressing cells. The threshold for statistical significant was set to *p<0.05* for all analyses.

## Supporting Information

Figure S1 **Expression of SLAM-R family by HHPHs and Huh-7 and HepG2 human HCC cell lines.** SLAM-Rs were stained with specific antibodies against SLAMF1 (CD150), SLAMF2 (CD84), SLAMF4 (CD224) and SLAMF6 (NTBA) (grey) or with matched isotype controls (empty). One of four independent experiments is shown.
(TIF)

Figure S2 **Expression of SLAMF3 assessed by flow cytometry analysis after transfection with vector coding for SLAMF3 or an empty vector (Mock) in Huh-7 and HepG2 cells.** SLAMF3 staining (in grey) is overlaid by negative control (in white). One of four independent experiments is shown.
(TIF)

Figure S3 **CFSE staining in SLAMF3-over-expressing cells and gated on SLAMF3^pos^ and SLAMF3^neg^ cells at 48 h compared to untreated cells (transfected with pBud free vector) and to CFSE baseline detected at 0 h.** One of four independent experiments is shown.
(TIF)

Figure S4 **Effect of SLAMF3 expression on the organization of the actin cytoskeleton.** Cells (Huh-7) were stained with phalloidin (rhodamine, red) and anti-SLAMF3 (FITC, green) and SLAMF3 positive (Huh-7-SLAMF3^pos^) and SLAMF3-negative (Huh-7-SLAMF3^pos^) cells were examined under the microscope. One representative of two independent experiments is shown.
(TIF)

Figure S5 **Evaluation of apoptosis in Huh-7 cells by annexin V/7-AAD staining.** At 48 h, dead cells (annexin V/7-AAD-positive) in SLAMF3-overexpressing cells and mock-transfected cells were counted. Results were presented as a dot plot (A) and the mean ± SD percentage of annexin V/7-AAD-positive cells (n = 3; statistical significance: ***p<0.005) (B); (C) the mean ± SD percentage of annexin V/7-AAD-positive cells per cent in SLAMF3^pos^ and SLAMF3^neg^ subpopulations of Huh-7 cells overexpressing SLAMF3 (n = 3; statistical significance: **p<0.01); (D) expression of BCL-2 family members in SLAMF3-overexpressing Huh-7 cells and mock-transfected cells. BAD (pro-apoptotic) and BCL-XL (anti-apoptotic) levels are shown as the results of one representative of two independent experiments.
(TIF)

Table S1 **Clinical parameters of HCC patients and METAVIR score.** NI: non infected; N: No; Y: Yes; sd: standard deviation; HBV: Hepatitis B virus; HCV: Hepatitis C virus; A: Activity; F: fibrosis; METAVIR score: A0 = no activity A1 = mild activity A2 = moderate activity A3 = severe activity; F0 = no fibrosis F1 = portal fibrosis without septa F2 = portal fibrosis with few septa F3 = numerous septa without cirrhosis F4 = cirrhosis.
(TIF)
