# Supplementary figures and images for: Identification of SLAMF3 (CD229) as an Inhibitor of Hepatocellular Carcinoma Cell Proliferation and Tumour Progression
Source: PLoS One. 2013 Dec 20;8(12):e82918. doi: 10.1371/journal.pone.0082918 (PMC3869749; doi:10.1371/journal.pone.0082918)

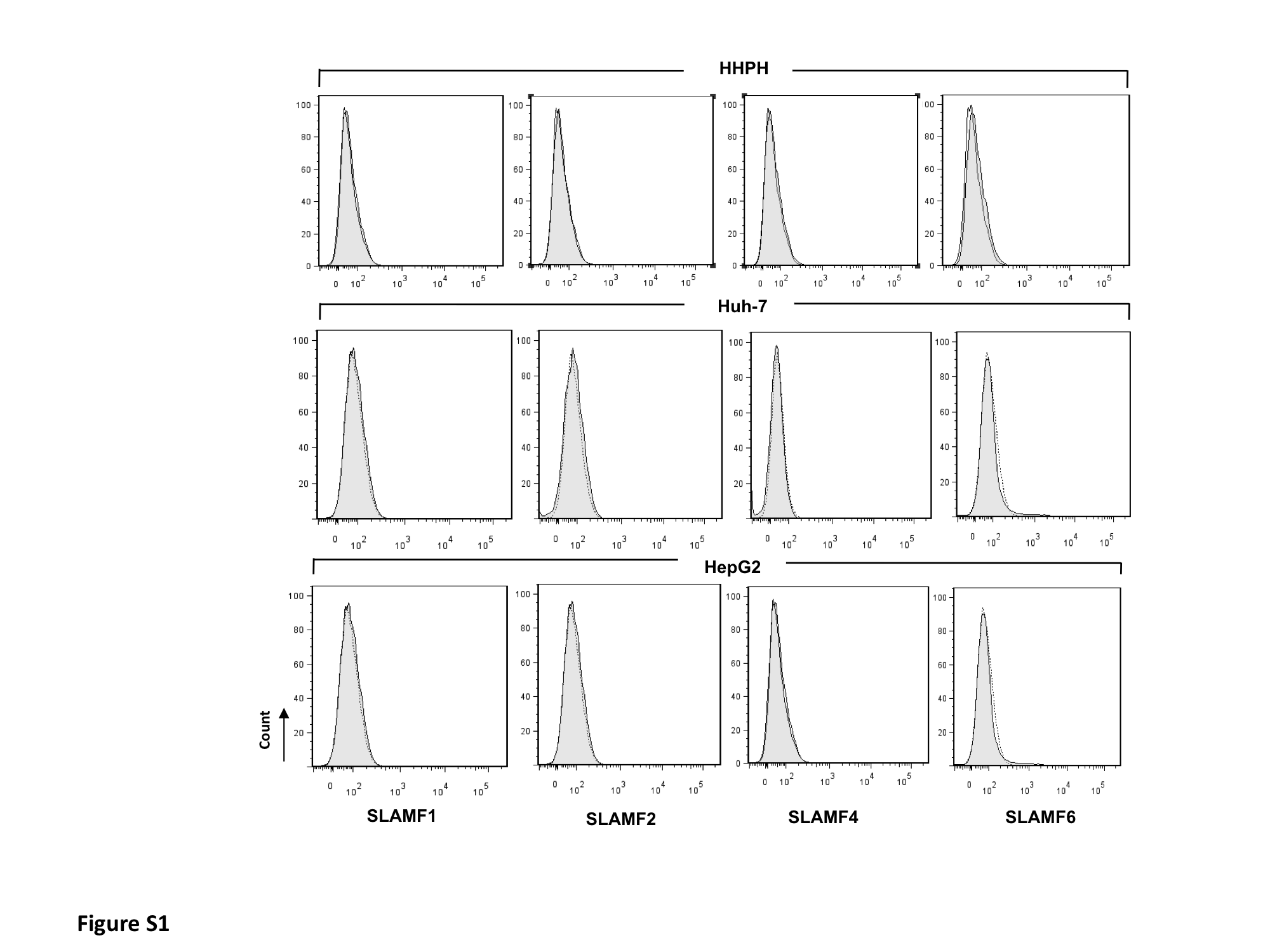

Supplement: Figure S1 — Expression of SLAM-R family by HHPHs and Huh-7 and HepG2 human HCC cell lines. SLAM-Rs were stained with specific antibodies against SLAMF1 (CD150), SLAMF2 (CD84), SLAMF4 (CD224) and SLAMF6 (NTBA) (grey) or with matched isotype controls (empty). One of four independent experiments is shown. (TIF) [file pone.0082918.s001.tif]

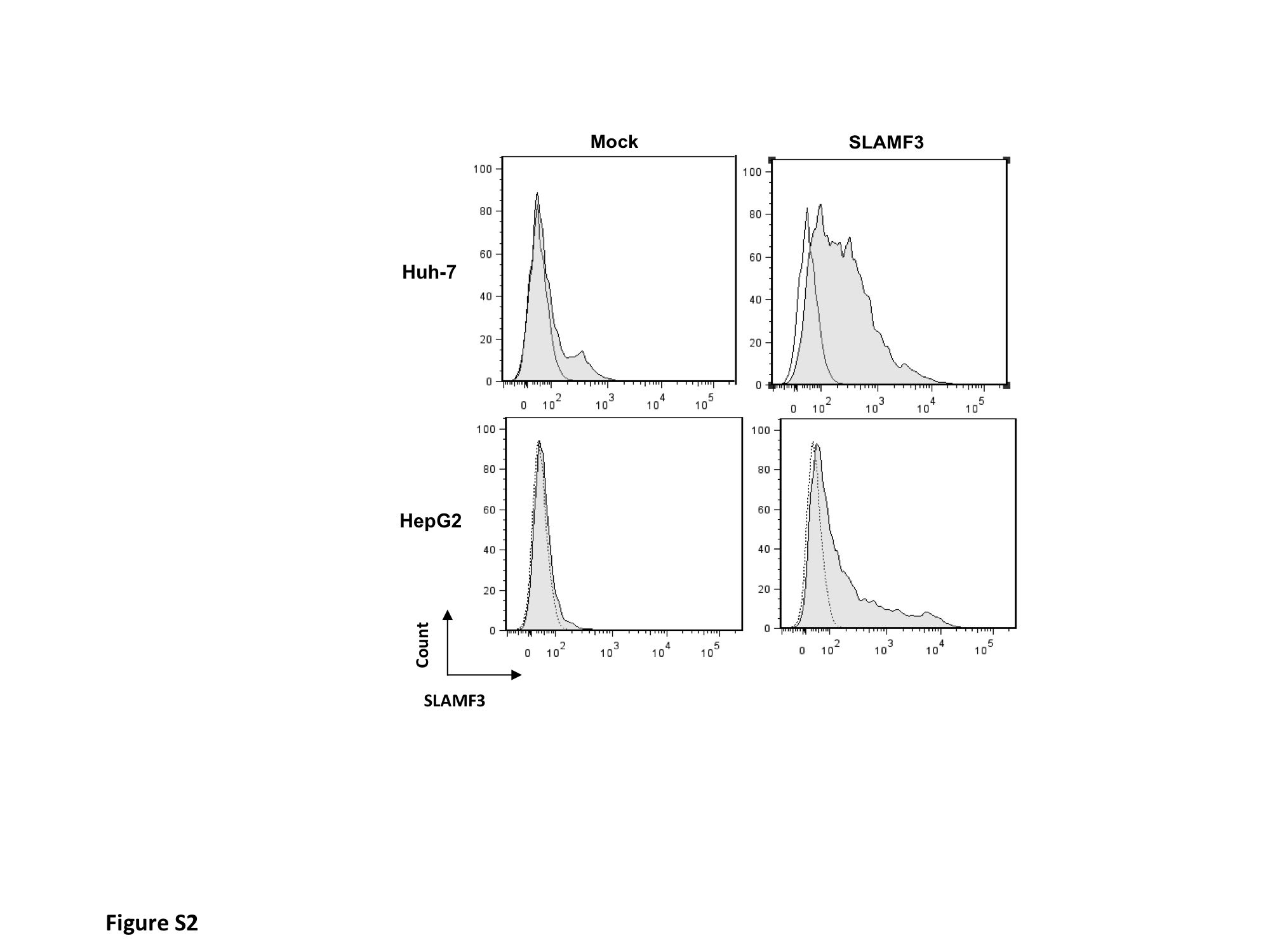

Supplement: Figure S2 — Expression of SLAMF3 assessed by flow cytometry analysis after transfection with vector coding for SLAMF3 or an empty vector (Mock) in Huh-7 and HepG2 cells. SLAMF3 staining (in grey) is overlaid by negative control (in white). One of four independent experiments is shown. (TIF) [file pone.0082918.s002.tif]

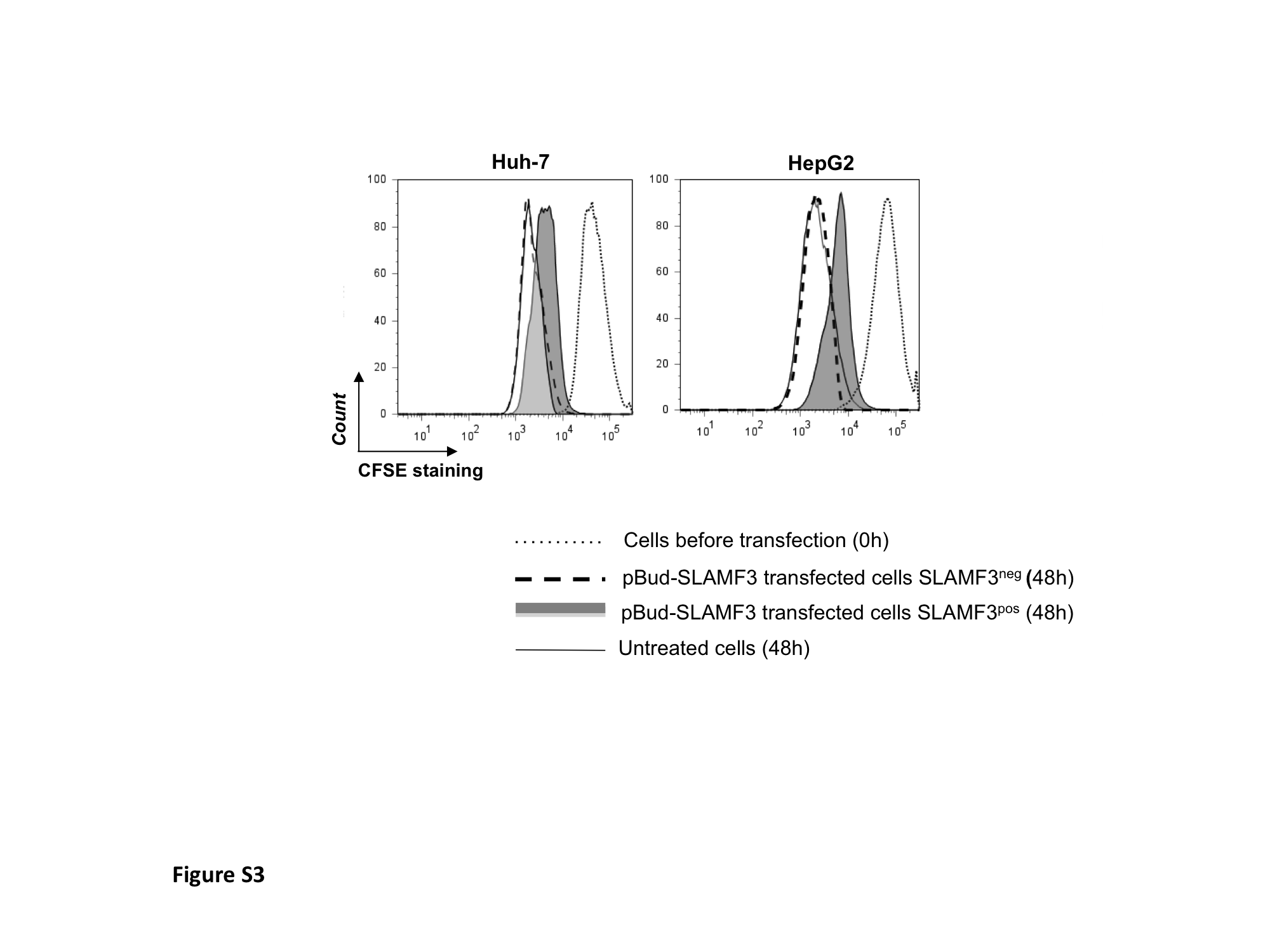

Supplement: Figure S3 — CFSE staining in SLAMF3-over-expressing cells and gated on SLAMF3pos and SLAMF3neg cells at 48 h compared to untreated cells (transfected with pBud free vector) and to CFSE baseline detected at 0 h. One of four independent experiments is shown. (TIF) [file pone.0082918.s003.tif]

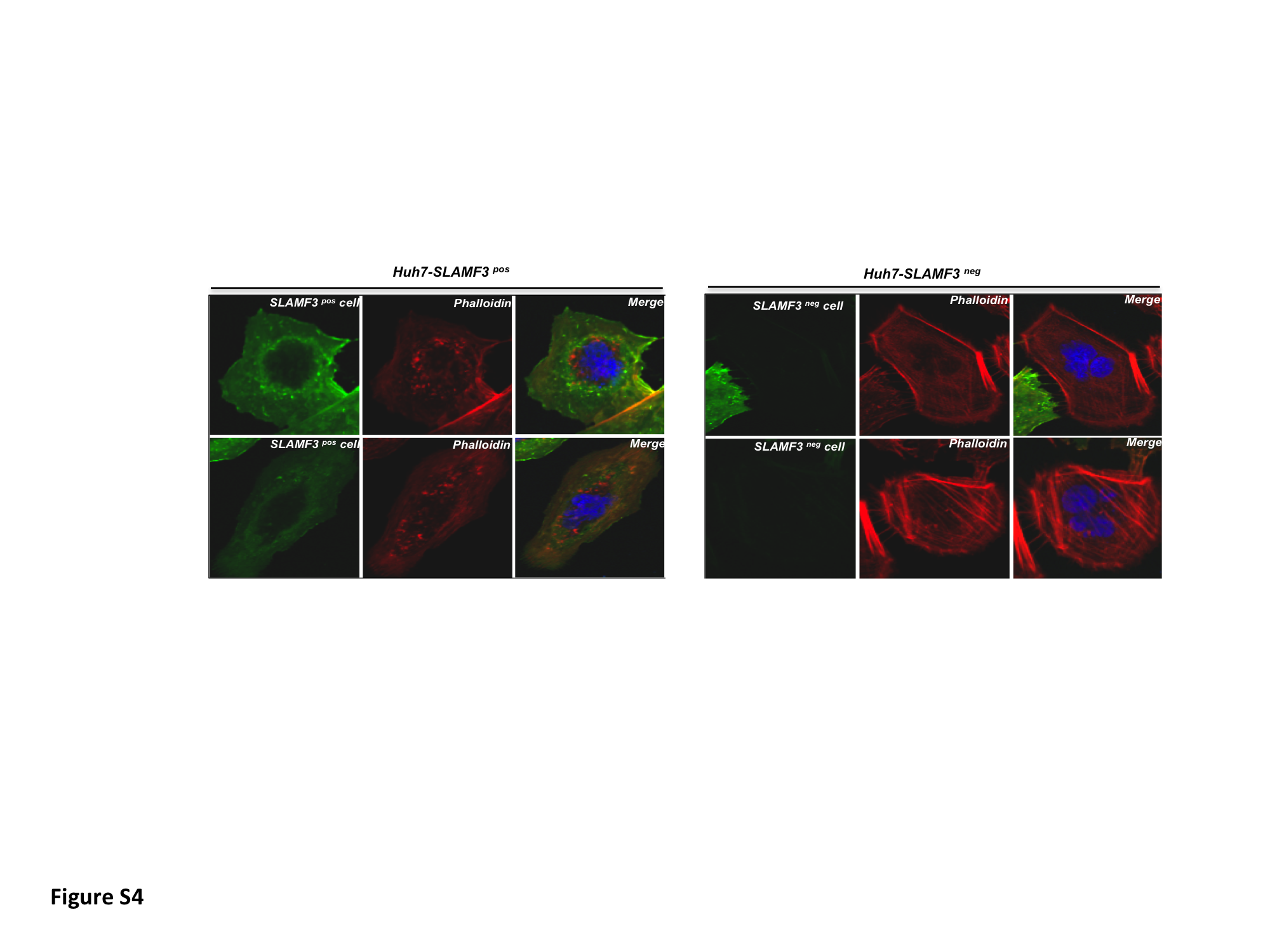

Supplement: Figure S4 — Effect of SLAMF3 expression on the organization of the actin cytoskeleton. Cells (Huh-7) were stained with phalloidin (rhodamine, red) and anti-SLAMF3 (FITC, green) and SLAMF3 positive (Huh-7-SLAMF3pos) and SLAMF3-negative (Huh-7-SLAMF3pos) cells were examined under the microscope. One representative of two independent experiments is shown. (TIF) [file pone.0082918.s004.tif]

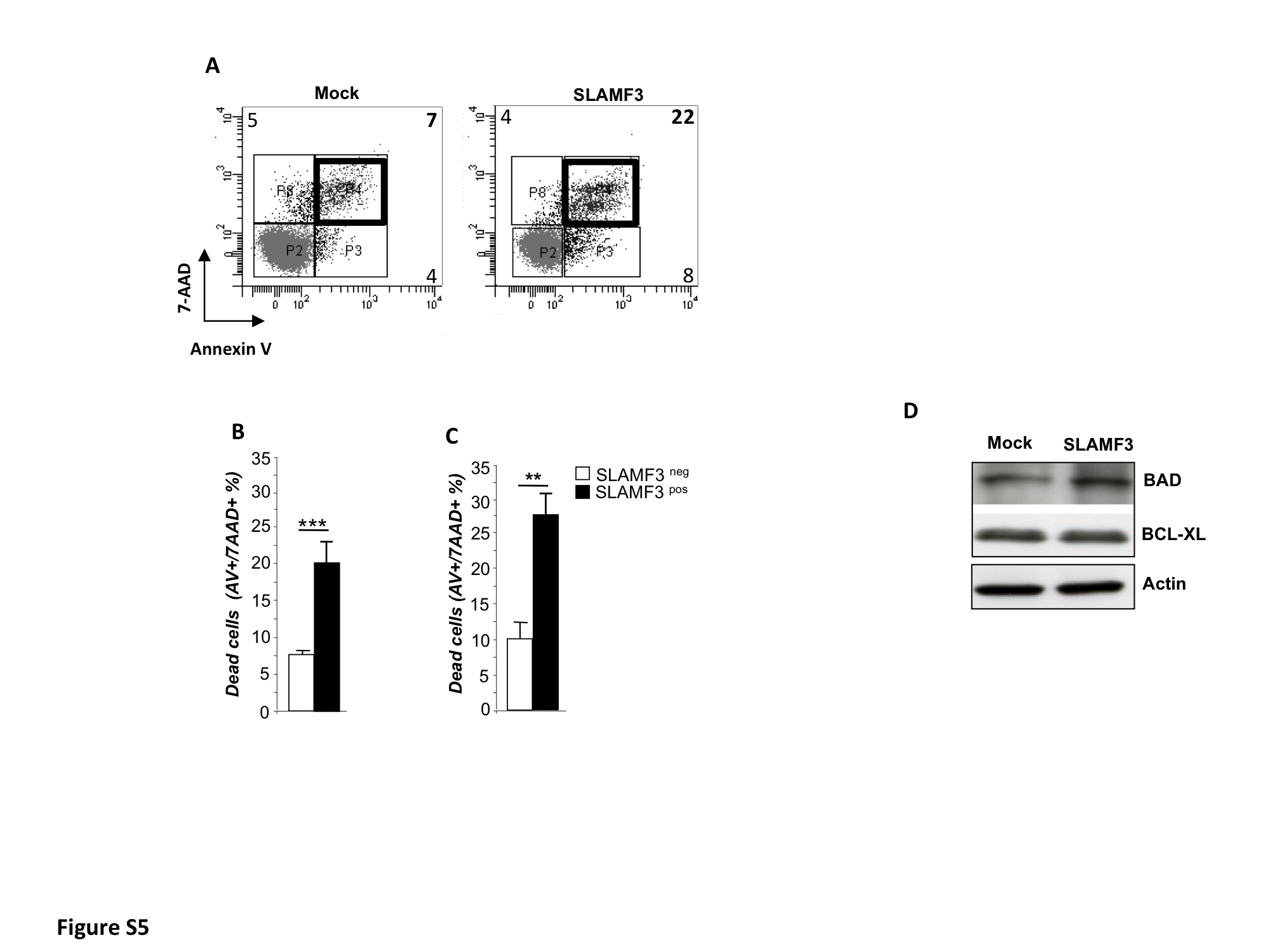

Supplement: Figure S5 — Evaluation of apoptosis in Huh-7 cells by annexin V/7-AAD staining. At 48 h, dead cells (annexin V/7-AAD-positive) in SLAMF3-overexpressing cells and mock-transfected cells were counted. Results were presented as a dot plot (A) and the mean ± SD percentage of annexin V/7-AAD-positive cells (n = 3; statistical significance: ***p<0.005) (B); (C) the mean ± SD percentage of annexin V/7-AAD-positive cells per cent in SLAMF3pos and SLAMF3neg subpopulations of Huh-7 cells overexpressing SLAMF3 (n = 3; statistical significance: **p<0.01); (D) expression of BCL-2 family members in SLAMF3-overexpressing Huh-7 cells and mock-transfected cells. BAD (pro-apoptotic) and BCL-XL (anti-apoptotic) levels are shown as the results of one representative of two independent experiments. (TIF) [file pone.0082918.s005.tif]

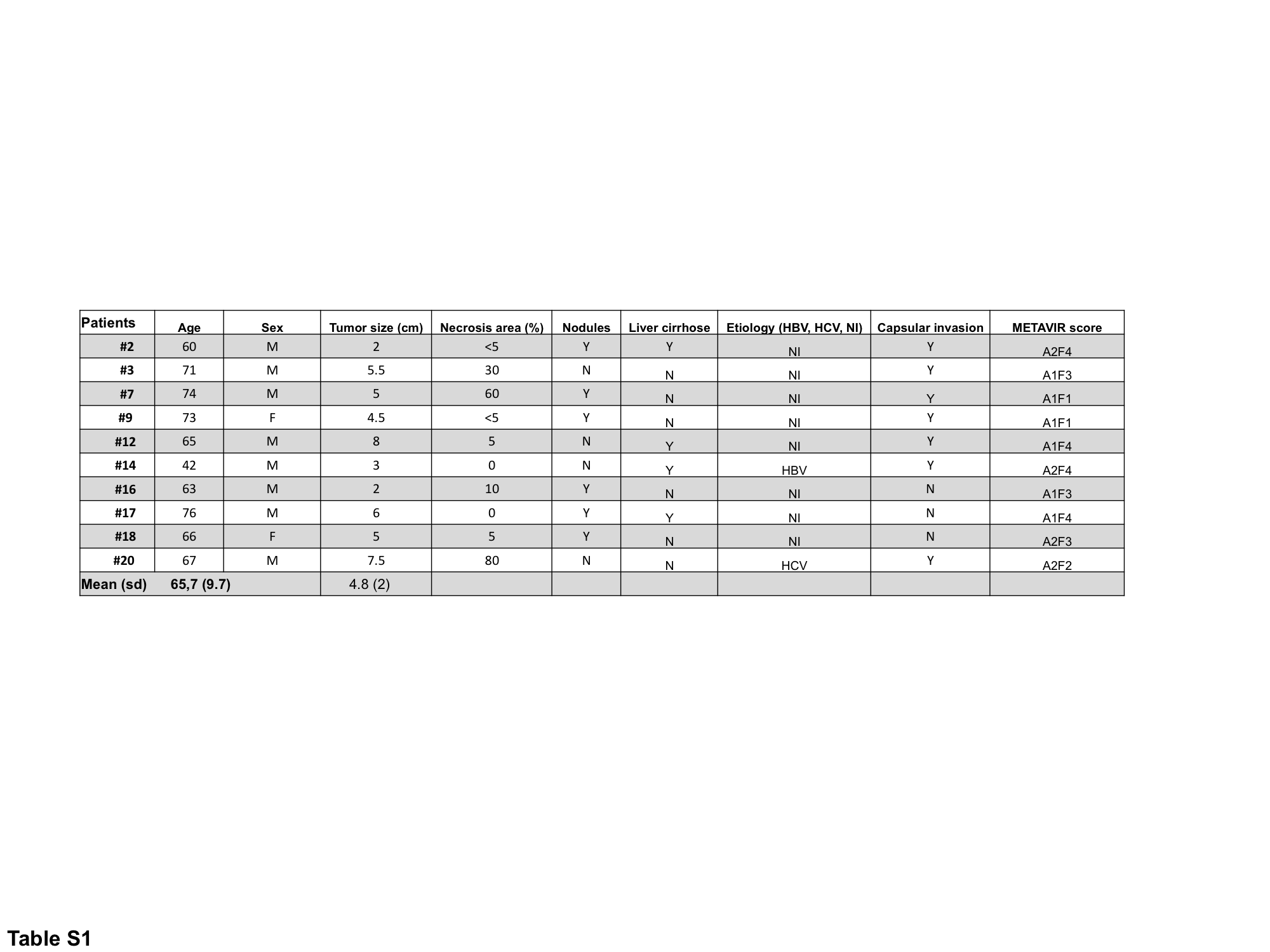

Supplement: Table S1 — Clinical parameters of HCC patients and METAVIR score. NI: non infected; N: No; Y: Yes; sd: standard deviation; HBV: Hepatitis B virus; HCV: Hepatitis C virus; A: Activity; F: fibrosis; METAVIR score: A0 = no activity A1 = mild activity A2 = moderate activity A3 = severe activity; F0 = no fibrosis F1 = portal fibrosis without septa F2 = portal fibrosis with few septa F3 = numerous septa without cirrhosis F4 = cirrhosis. (TIF) [file pone.0082918.s006.tif]
